# First Case Report of Turcot Syndrome Type 1 in Colombia

**DOI:** 10.1155/2012/356384

**Published:** 2012-12-18

**Authors:** Vallejo Dora, Garnica Diego, Bonilla Rómulo, Olaya Natalia

**Affiliations:** ^1^Human Genetics Research Group, Industrial University of Santander (UIS), Bucaramanga, Colombia; ^2^Hospital Universitario de Santander (HUS), Universidad Industrial de Santander (UIS), Bucaramanga, Colombia; ^3^Grupo de Patología Oncológica, Instituto Nacional de Cancerología, ESE, Bogotá, Colombia

## Abstract

Turcot syndrome is an autosomal recessive disorder clinically characterized by the occurrence of primary tumors of the central nervous system and adenomatous colonic polyps during the first or second decades of life, with a spectrum of clinical features such as “café-au-lait” spots, axillary freckling, and hyperpigmented spots. Currently its prevalence globally and in Colombia remains unknown. We present the case of a 20-year-old male with a clinical presentation of both glioblastoma multiforme and multiple adenomatous colonic polyps. The molecular genetics study revealed a mutation in Kras^Asp12^ gene and altered expression of HMSH2 and HMSH6 proteins encoded by the DNA mismatch repair genes in two of the colonic polyps. Even though this clinical presentation may suggest a shorter survival rate, this patient is still alive after seven months of treatment. A literature review complements this report.

## 1. Introduction

Turcot syndrome (OMIM: no. 276300, mismatch repair cancer syndrome) is an autosomal recessive disorder [1] characterized by concurrent presentation of a primary tumor of the central nervous system and adenomas or colorectal carcinoma [2], during the first or second decades of life, with a spectrum of clinical features such as “café-au-lait” spots, axillary freckling, and hyperpigmented spots [3]. It is caused by homozygous or heterozygous mutations in the mismatch repair system (MMR) genes: MLH1, MSH2, MSH6, or PMS2, locus on 3p22.2, 2p21, 2p16.3, and 7p 22.1, respectively [4]. Global prevalence is unknown.

## 2. Case Report

**Figure 1:**
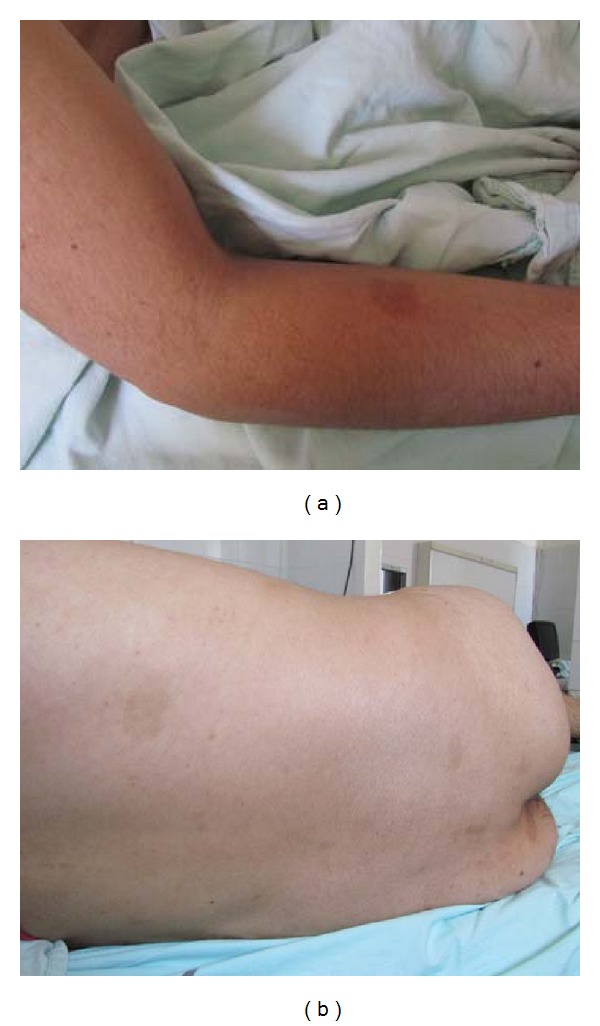
Photograph of the patient skin. It shows multiple café- au- lait macules on the anterior arm side (a) back of 5 × 3 cm and buttocks (b).

**Figure 2:**
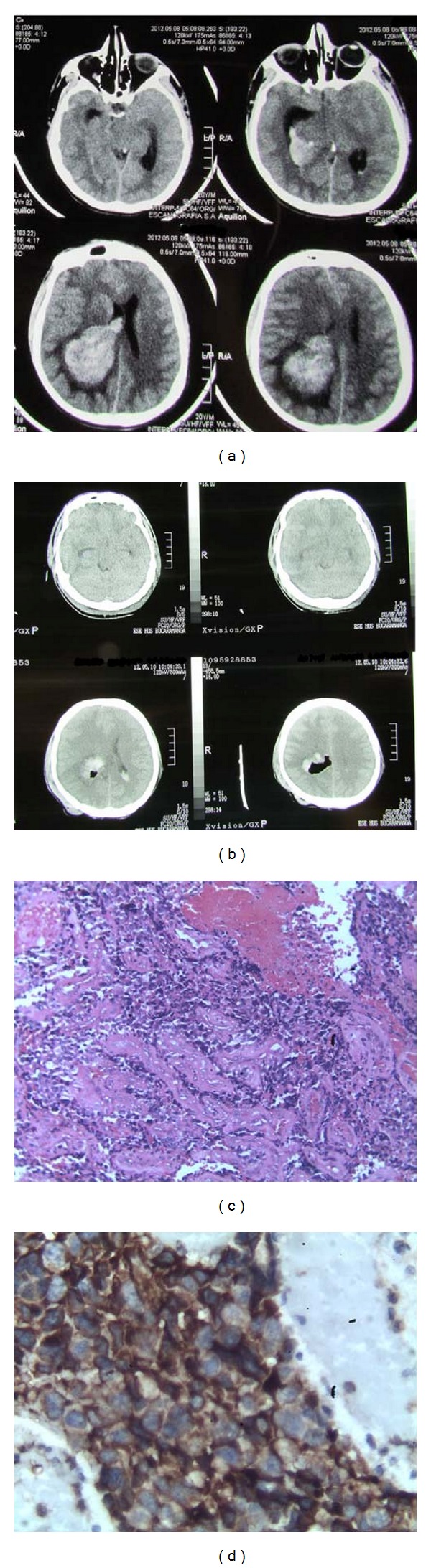
The brain CT. Parietotemporal tumor and intraparenchymal hemorrhage associated left ventricular drainage system and midline shift (a) and after resection demonstrates no residual tumor and a resolve mass effect (b). Histopathology shows high mitotic index and multinucleated giant cells. Hematoxylin and eosin stain (c). Immunohistochemistry confirmed the glial origin and high proliferation rate and PAFG positive (d).

A 20-year-old male presented with tonic-clonic seizure and loss of consciousness. He had presented headache and vomiting for the previous 20 days. Both clinical symptoms were compatible with intracranial hypertension. He had history of weakness, weight loss, and other constitutional symptoms, and family history of consanguinity. During the clinical examination, he was fully awake and oriented; a motor deficit in the right side was noticed. On his skin, there were multiple *café-au-lait* spots and areas of hyperpigmentation (Figures 1(a) and 1(b)). Brain CT showed that he had a 4 × 3 cm intraparenchymal hemorrhage with mass effect of the left parietotemporal side. He underwent left parietotemporal craniotomy, gross total resection of the tumor, and abscess drainage (Figures 2(a) and 2(b)).

**Figure 3:**
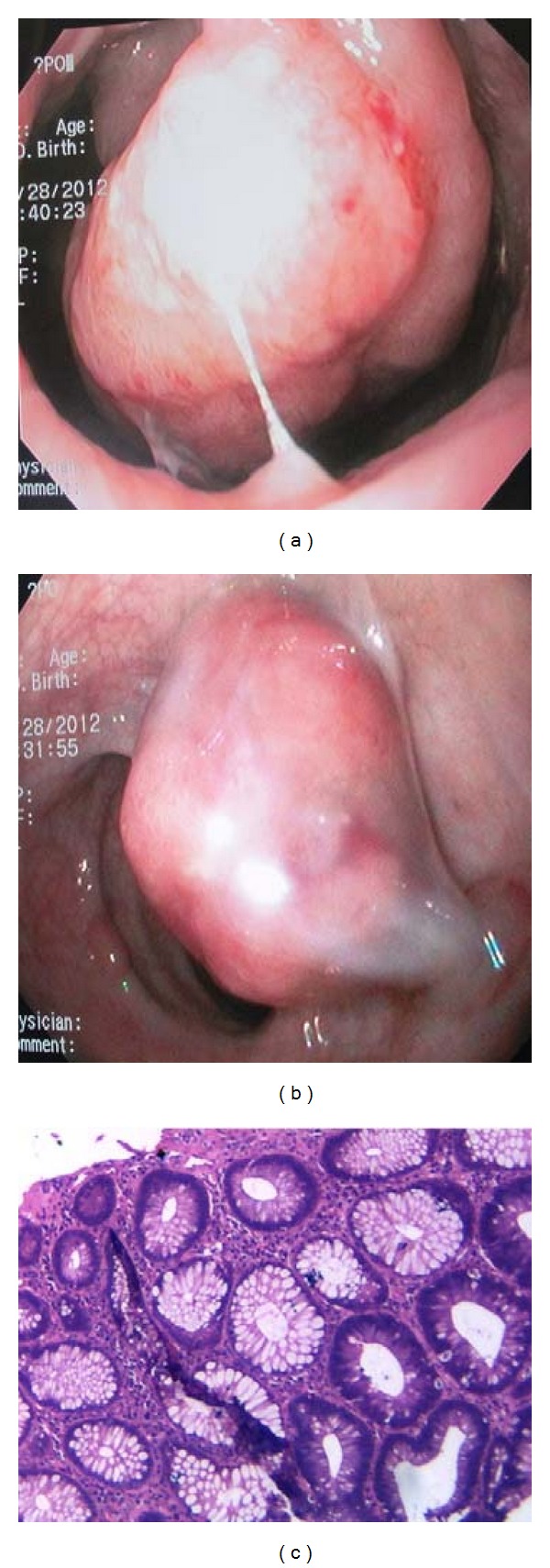
Colon endoscopy demonstrates tubulovillous adenomatous polyps, a sessile polyp 40 mm with adenomatous aspect in rectum (a) and in sigmoid colon (b).

**Figure 4:**
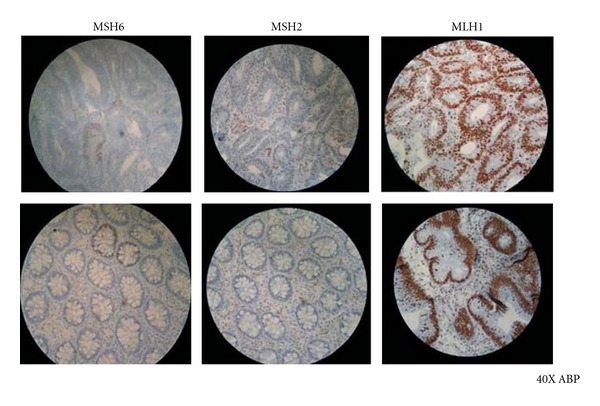
IHC evaluation of complex protein expression MMR. Reactivity was observed in the presence of brown coloration in this case there is not involvement of the MLH1 protein, while exhibiting an alteration in the expression of proteins MSH2 and MSH6 as seen in the images of blue coloration polyp 1.

During hospitalization, he was also suffering of lower gastrointestinal tract bleeding. The colon endoscopy revealed 25 colorectal polyps. Histologic analysis of the brain lesion demonstrated a malignant proliferation of pleomorphic and hyperchromatic cells in a background of necrosis. Immunohistochemistry confirmed the glial origin and high proliferation rate. (PFAG positive, high Ki-67 index) (Figures 2(c) and 2(d)). The tumor was diagnosed as glioblastoma multiforme. Some of the lesions found during colon endoscopy were tubulovillous adenomatous polyps with high-grade dysplasia (Figures 3(a), 3(b), and 3(c)). Adenomatous polyps were negative for HMSH2 and HMSH6 [5] protein expression, which were evaluated by immunohistochemistry (Figure 4). HMLH expression was preserved. Quantitative PCR was performed for evaluating common mutations of the Kras gene. Kras^Asp12^ mutation was detected [6].

## 3. Discussion

**Figure 5:**
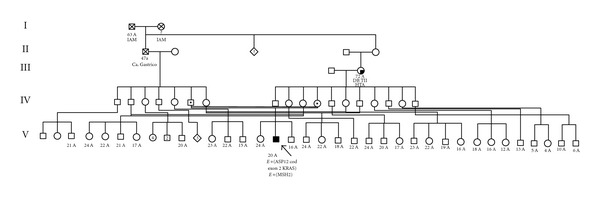
Pedigree of the family of the patient illustrates consanguinity in the second generation.

Turcot syndrome is one of the diseases belonging to the hereditary nonpolyposis colorectal cancer syndrome (HNPCC), also known as Lynch syndrome. HNPCC is characterized by an inherited mutation in one of four DNA mismatch repair genes, (MLH1, MSH2, MSH6, and PMS2). These mutations predispose patients to the development of colon cancer and brain tumors, more commonly medulloblastoma. The diagnosis of HNPCC is based on a combination of clinical and genetic criteria. There are two kinds of Turcot syndrome. Patients with Turcot syndrome type 1, they have been described carrying mutations in the MLH1, MSH2, and the PMS2 genes, but chiefly are found to have mutations in MLH1 and PMS2 MMR gene products are in charge of repair base mismatches and insertion deletion loops during DNA replication. The gene mutations in MMR produce an environment of genetic instability in small repeated gene sequences called microsatellites. When somebody inherits a germline mutation in one allele, a second somatic mutation can occur, allowing for the initiation of tumors. Turcot syndrome type 2, usually found in FAP, presents with innumerable adenomatous polyps [7].Central nervous system brain tumors occur in both types; type 1 consists of astrocytoma or glioblastoma, and type 2 consists of medulloblastoma [8]. In Latin America, there are four cases of Turcot syndrome reported. Our patient had the phenotypical and pathological findings of Turcot syndrome. As he is the issue of a family with consanguinity (Figure 5), this enabled a precautionary management that improved patient and siblings prognosis. It is imperative to complete the molecular testing and genetic counseling for early diagnosis and preventive management in the family.
